# The HOME (home monitoring of high-risk pregnancies) study: a study protocol for an observational study of a telemedicine-assisted follow-up at home vs. hospitalization

**DOI:** 10.3389/fgwh.2025.1599153

**Published:** 2025-07-14

**Authors:** Åsa Henning Waldum, Aase Serine Devold Pay, Gunvor Aasbø, Vinod Kumar Mishra, Meryam Sugulle, Anne Cathrine Staff

**Affiliations:** ^1^ Division of Obstetrics and Gynaecology, Oslo University Hospital, Oslo, Norway; ^2^ Department of Gynecology and Obstetrics, Vestre Viken Hospital Trust, Bærum, Norway; ^3^ Faculty of Health Sciences, Oslo Metropolitan University, Oslo, Norway; ^4^ Department of Public Health Science and Interdisciplinary Health Science, Faculty of Medicine, University of Oslo, Oslo, Norway; ^5^ Department of Finance and Resource Management Unit, Oslo University Hospital, Oslo, Norway; ^6^ Institute of Clinical Medicine, Faculty of Medicine, University of Oslo, Oslo, Norway

**Keywords:** pregnancy, high-risk, hospitalization, fetal membranes, premature rupture, home care services, telemedicine, safety, patient reported outcome measures

## Abstract

**Background:**

Pregnancies at high risk for adverse health outcomes for mother and offspring often require long-term antenatal hospitalization and/or frequent outpatient visits. We have developed a telemonitoring home care service for high-risk pregnancies that has been integrated into the Electronic Patient Journal System of our department. We will compare clinical safety, patient-reported outcome measures, and use of healthcare resources compared to standard practice for hospital admissions and/or outpatient visits.

**Method:**

The home monitoring of high-risk pregnancies study is an ongoing observational study. Eligible women with a pregnancy requiring intensified obstetric follow-up (e.g., preterm premature rupture of membranes, hypertensive disorders of pregnancy, or a previous adverse obstetric outcome) are offered study inclusion to either standard care at the hospital or the home telemonitoring group, depending on available home monitoring equipment. Pregnant women included for home monitoring will be telemonitored according to relevant clinical practice for inpatients, including the use of cardiotocography, blood pressure monitoring, C-reactive protein, and temperature measurement, and they will provide self-registration of relevant clinical symptoms. A telecare patient communication system will prompt rapid contact with the hospital in the case of unfavorable registered clinical parameters or subjective symptoms. The home telemonitored women will attend hospital visits for fetal ultrasound assessment at individually assigned intervals. Patients undergoing in-hospital care will serve as the control group in this study and receive standard care. The primary outcome is a composite of severe maternal and perinatal adverse outcomes (sepsis, eclampsia, cerebral hemorrhage, acute respiratory distress syndrome, liver rupture, pulmonary embolism, amniotic fluid embolism, hemolysis, elevated liver enzymes, low platelets, HELLP without hemolysis, and disseminated intravascular coagulation), including fetal or neonatal mortality, maternal mortality, and signs of severe organ damage. Secondary outcomes include other adverse maternal and fetal/neonatal health outcomes, patient-reported outcomes, and economic cost analyses.

**Discussion:**

The implementation of a home care service for women with high-risk pregnancies requiring intensified surveillance is expected to be equally safe and more comfortable and convenient for the women, with lower economic costs.

**Clinical Trial Registration:**

Clinicaltrial.gov, NCT05763069.

## Introduction

The surveillance of mother and fetus is crucial for detecting early warning signs of maternal and/or fetal distress in pregnancies with an increased risk for adverse maternal and/or fetal outcomes (1, 2). If worrying maternal and/or fetal signs during pregnancy are not recognized and treated appropriately, the risk of mortality and morbidity is increased for both (3, 4). The lack of appropriate treatment may potentially have lifelong health consequences for the mothers and infants who survive. Surveillance in high-risk pregnancies aims to optimize early diagnosis, treatment, and timing for delivery in order to optimize health outcomes (3, 4). Specialized obstetric surveillance, including cardiotocography (CTG; registration of the fetal heart rate pattern and uterine contractions), blood pressure, and urine and blood analyses, often requires antenatal hospitalization and/or frequent outpatient visits until delivery.

High-risk pregnancies that often require intensified surveillance include those with hypertension during the pregnancy (e.g., chronic hypertension, gestational hypertension, and preeclampsia) and those with preterm premature rupture of membranes [pPROM; preterm premature (prior to 37 weeks of pregnancy) rupture of fetal membranes prior to onset of labor], the latter conferring a risk of preterm birth and/or chorioamnionitis, with increased risk of maternal and/or fetal death. In addition, some women with a history of adverse obstetric outcomes have a propensity for recurrence (e.g., previous intrauterine fetal demise, fetal growth restriction, or severe preeclampsia), thereby requiring intensified surveillance in the following pregnancy to optimize maternal and offspring health outcomes.

Recent technological development has generated new opportunities for health surveillance outside the hospital, i.e., at home. Home monitoring systems for high-risk pregnancies, with the opportunity for patient registration of CTG, have not been tested or established in most countries, including Norway. After the planning of the home monitoring of high-risk pregnancies (HOME) study, two articles reporting on outcomes after home monitoring of high-risk pregnancies have been published: one retrospective study of 8 years of practice in Denmark (5) and one randomized controlled trial from the Netherlands (6). Both concluded that home monitoring may be a safe alternative to inpatient hospital care for selected women with high-risk pregnancies. Home monitoring of blood pressure in pregnancy poses challenges, as blood pressure can rise rapidly during pregnancy. Monitoring of blood pressure at home in pregnant women with gestational hypertension has, however, demonstrated better compliance with guidelines, likely lowering the burden on public health resources (7).

Any change in clinical practice needs to be thoroughly evaluated, including an evaluation of clinical outcomes. Thus, in this study, clinical safety, patient-reported outcomes, and healthcare economics following the introduction of home monitoring in high-risk pregnancies will be investigated. Ultimately, personalized follow-up with more telemonitoring opportunities and flexibility may enable the much in-demand specialist resources at obstetric departments (e.g., midwives and obstetricians) to focus on the patients with the greatest need for in-hospital follow-up.

The hypothesis in this clinical study is that home monitoring in selected high-risk pregnancies does not increase the risk of severe adverse maternal or fetal outcomes for women and their fetuses compared to similar pregnancies being followed up through hospitalizations and/or frequent outpatient visits. We also anticipate increased patient-reported satisfaction in line with positive patient expectations from general technology-enabled care solutions (8) and findings from our own preclinical expectation study related to the HOME study (9). Finally, we expect that the freeing up of hospital beds will reduce hospital costs and thereby result in a positive health economic assessment, despite the increased device costs and reorganization of the hospital’s follow-up system.

## Materials and methods

### Study setting

The observational HOME study has been carried out in southeastern Norway, primarily at Oslo University Hospital (OUH) in Norway's capital, Oslo, which has the largest Obstetric Department in Norway, with 8,000 deliveries annually. Another hospital in the region, Drammen Hospital, with 2,000 deliveries annually, will also recruit some patients to the study.

Norway offers pregnant women free-of-charge antenatal, delivery, and postpartum care. Women deemed at high risk for adverse pregnancy outcomes, either due to current pregnancy complications or previous adverse obstetric outcomes, are offered intensified follow-up according to a national expert guideline (3).

The observational study protocol for this study was posted on ClinicalTrials.gov in 2023 (10 March 2023: ref: NCT05763069). The first woman was recruited to the hospital-monitored group in December 2022. The first woman monitored at home was recruited in June 2024, following a 2-year period of study adaptation due to General Data Protection Regulation (GDPR) issues and technology solution developments for the transfer of home-registered data to the hospital's electronic patient journal systems.

### User involvement

The HOME study has involved user groups in the planning of the study, including the local hospital advisory user group and the Norwegian SIDS and Stillbirth Society. The latter society also provides advisory help during all trial phases, as a member of the steering group and as a non-academic named collaborator in the HOME study grant obtained from the Research Council of Norway (ref. 326650).

Prior to recruiting women to the HOME study, a qualitative study was conducted to address user perspectives on home-based telemonitoring as an alternative to hospital admissions and/or frequent outpatient visits. Hospitalized women with ongoing high-risk pregnancies, women with a history of stillbirth in a previous pregnancy, midwives, and obstetricians (total *n* = 21) were interviewed (9). The results showed that the participants not only acknowledged the benefits and potential of home monitoring but also highlighted their concerns regarding clinical safety and responsibility. Home monitoring of high-risk pregnancy was problematized in terms of personal/individual factors, such as the need for personalized training, eligibility assessments of individuals, and empowerment to undertake and cope with a more active patient role. A sense of shared responsibility was regarded as crucial to maintain safety, particularly when acute or critical situations emerge (9).

In line with these user expectations, published in 2022, we developed user instruction protocols for home monitoring training while the patient is still in the hospital. We developed clinical questionnaires regarding patient-hospital communication using smartphone platforms that were adapted to the specific pregnancy complications in the study (e.g., hypertensive disorders vs. pPROM). We also updated the instruction protocols for supervising healthcare staff in the clinical setting of home monitoring, including check lists and selection processes to identify pregnancies deemed clinically relevant for home monitoring, daily follow-up systems, electronic patient journal documentation, and the use of the most appropriate public reimbursement classifications (as none have hitherto been specifically developed for home monitoring of high-risk pregnancies).

### Participant eligibility criteria

The patients eligible for participation in the study are ≥18 years old, Norwegian-speaking, singleton pregnant women requiring intensified follow-up after at least one initial comprehensive hospital assessment. The following indications for surveillance may be relevant for home monitoring (Table 1): hypertensive disorders (chronic hypertension, gestational hypertension, and/or preeclampsia), pPROM, and previous adverse obstetric outcomes (e.g., fetal death, severe preeclampsia, preterm delivery, and/or fetal growth restriction). Exclusion criteria for home monitoring include a clinical imminent health risk to the mother and/or fetus, including (but not restricted to) non-reassuring CTG, blood pressure, infectious or clinical signs or symptoms, and/or more than 1 h travel to the hospital. Women with an expected delivery or induction of delivery within a short period will not be recruited [e.g., preeclampsia at term (≥37 weeks), prelabor rupture of membranes (PROM) ≥37 weeks, or pPROM with indication for rapid delivery (e.g., discolored amniotic fluid, suspicion of chorioamnionitis or other severe infections, or pathological CTG)]. Insufficient support at home and a lack of understanding of the relevant HOME study's technical devices (to be tested before departure from the hospital) are also exclusion criteria for being offered home monitoring.

**Table 1:** Clinical definitions of the inclusion criteria for home monitoring of high-risk pregnancies.

	Inclusion criteria	Additional definitions
1	Hypertensive disorders of pregnancy	
-	✓ Chronic hypertension	✓ Hypertension (systolic blood pressure ≥140 mmHg and/or diastolic blood pressure ≥90 mmHg) identified before or during the first 20 weeks of the present pregnancy
	✓ Gestational hypertension	✓ New-onset hypertension (defined above) at ≥20 gestational weeks during the present pregnancy
	✓ Preeclampsia	✓ Gestational hypertension (defined above) in addition to new-onset proteinuria or at least one other maternal sign of organ dysfunction according to International Society for the Study of Hypertension in Pregnancy criteria from 2018 (4)
2	Preterm premature rupture of membranes	✓ Rupture of membranes at <37 gestational weeks ✓ No present uterine contractions ✓ Cephalic presentation
3	Previous adverse obstetric outcomes	✓ Intrauterine fetal death, severe preeclampsia (e.g., delivery prior to week 37 and/or severe fetal growth restriction) ✓ Preterm delivery (prior to week 37) ✓ Fetal growth restriction

### Recruitment procedure

**Figure 1:**
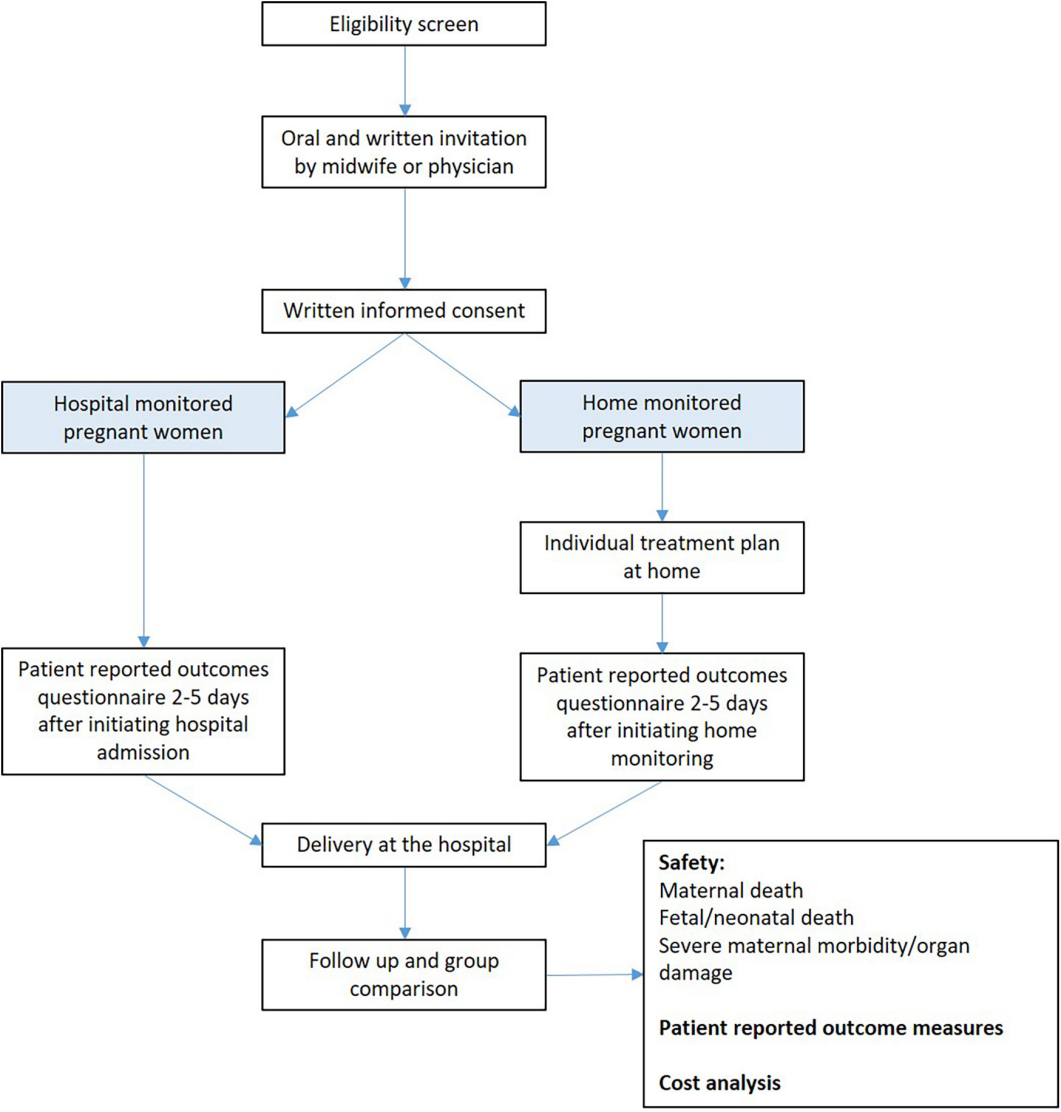
Flowchart of the selection of participants for home monitoring of high-risk pregnancies.

A senior obstetrician identifies eligible pregnant women (followed up at the hospital) for home monitoring, according to the aforementioned medical inclusion and exclusion selection criteria, using a preprinted checklist. Eligible women are thereafter invited and informed by a trained research midwife, who has sufficient time with each woman to answer questions and explain the practical procedures of monitoring either in the hospital or at home. Oral and written information is provided, and written informed consent is thereafter obtained from eligible women willing to participate in the study. The timeline for participation in the study is shown in Figure 1. The midwife will, prior to study inclusion, also assess whether the woman has adequate support at home and sufficient technical understanding, including testing the patient's capability to use the specific information technology (IT) solutions necessary for home monitoring.

The allocation of women to the regular follow-up or home monitoring groups is based on technical equipment availability, as explained below.

#### Hospital-monitored group details

The women in the hospital-monitored group are women who met the inclusion criteria (Table 1) and were recruited to the study from 2022 to 2024, prior to the final approvals and testing of all the technical devices for home monitoring in the clinic. In addition, some of the women eligible for home monitoring participation from 2024 will be recruited to the hospital-monitored group for reasons unrelated to the inclusion and exclusion criteria (e.g., unavailable home monitoring devices). The hospital-monitored women are treated and monitored according to standard care.

#### Home-monitored group details

The women included in the home monitored group are women who met the inclusion criteria (Table 1). They were recruited to the study from 2024, following the final regulatory study approvals and integration of the technical home monitoring and reporting devices into the hospital electronic patient journal (EPJ) system. These women will be provided a training session with the research midwife in order to operate the medical devices they will use during home monitoring. The training will be conducted using standardized written and oral instructions relevant for the main diagnostic groups (e.g., hypertension or pPROM). The personalized follow-up plan includes instructions for how to contact the hospital 24/7 and how to be admitted when needed.

During home monitoring, women will respond to a checklist (Supplementary 1) on an application (app) (MyDignio). This eHealth application will be downloaded to the patient's smartphone during their hospital stay. The app has a direct connection to the electronic patient journal system at the hospital. Dignio Connected Care is approved by the South-Eastern Norway Regional Health Authority (the region where the HOME study is carried out) as a device for general patient home monitoring in the region. The researchers in the HOME study have developed relevant questions about symptoms and objective measures of the pregnant woman's health according to the indication for inclusion (e.g., pPROM or hypertensive disorders of pregnancy) (Supplementary 1).

The women included in the home monitoring group due to hypertensive pregnancy disorders will be asked questions in the app about symptoms related to increased severity of the disease (e.g., headache, swelling, epigastric pain, visual symptoms, fetal movements) and asked to enter their blood pressure values (recorded with a validated blood pressure device available for this study from the hospital) and results from the urine protein test.

For women included in the home monitoring group due to pPROM, the MyDignio app includes questions related to infection and premature birth (e.g., fever, pain, amniotic fluid color, and fetal movements), and objective markers of infection and fetal wellbeing signs are registered [e.g., temperature recording (measured using a study-provided device) and C-reactive protein test (CRP)]. According to hospital guidelines, vaginal and urine tests for bacterial infection will be performed before the start of home monitoring.

The women included due to previous adverse obstetric events will be asked questions about fetal movements in the MyDignio app. They may also be assigned a mix of questions from the other described risk groups, as indicated by the senior obstetrician.

The study-specific feedback given to the patient in the MyDignio app in response to the registered answers and registrations of symptoms and signs are detailed in Supplementary 1. The home-monitored group is, in addition to daily MyDignio app reports, instructed to perform CTG (see details below) according to the clinical indications set by the senior obstetrician. Many home-monitored patients will also be scheduled for an ultrasound investigation once a week at the hospital to assess fetal wellbeing and growth and amniotic fluid volume. Women with pPROM will, in addition, be scheduled for vaginal and urine testing of bacterial infection and infection status (CRP and leucocytes) and CTG at individually assigned intervals. Likewise, women with hypertensive disorders will be scheduled for blood samples and urine testing (until proteinuria is established) at individually assigned intervals.

#### Technological solutions for telemonitoring

The technological solutions for registering clinical data during home monitoring include the Nemo healthcare system (Veldhoven, the Netherlands) for home CTG monitoring. In addition, a web-based communication platform will be used by the home-monitored women (Dignio Connected Care, Norway) (10), which is accessible by the women through MyDignio (for patient symptoms and clinical signs) and by the healthcare providers (through DignioPrevent). Both systems are European conformity (CE)-licensed medical devices. The Nemo healthcare system has been evaluated as acceptable by users and has been found to improve telemedical understanding (11).

The home-monitored patients in the present study will record CTG at individually assigned intervals with an electrocardiogram-based device placed on the abdomen (Nemo Fetal Monitoring System), recording fetal heart rate patterns, uterine contractions, and maternal pulse. The signals are sent wirelessly to a CTG-receiver (a tablet; iPad, Apple) that transfers the signals through an encrypted data channel to the Milou Real-Time Server software (Medexa, Sweden). The CTG traces are visually interpreted by healthcare professionals at the hospital, according to international guidelines (12). These fetal and uterine signals are not visible on the user’s iPad screen (the pregnant woman), and are only visible to the healthcare staff at the hospital. Two recent studies have, after the planning of the HOME study, concluded that the use of such a home CTG system is feasible for women with pregnancy complications (5, 6).

The Microlife WatchBP Home monitor used in the present study has been found to be valid for monitoring blood pressure in the general and the pregnant populations (13–15). Appropriate cuff widths are used according to arm circumference (13). For women with pPROM, CRP is analyzed at home by the woman using the QuickRead go Instrument, which has shown acceptable agreement with reference methods (16, 17). Rectal temperature is measured by the Microlife MT 800 Digital Thermometer.

### Outcome measures

The primary outcome variable is a composite of severe adverse events, as detailed in Table 2. These include maternal mortality, severe maternal morbidity/organ damage [e.g., sepsis, eclampsia, cerebral hemorrhage, acute respiratory distress syndrome, liver rupture, pulmonary embolism, amniotic fluid embolism, hemolysis, elevated liver enzymes, and low platelets (HELLP), HELLP without hemolysis (ELLP), and/or disseminated intravascular coagulation (DIC)], and fetal or early neonatal death. The primary composite outcome will be thoroughly evaluated by the Diagnostic Advisory Group (DAG). The members of the DAG include three senior obstetricians, one senior pediatrician, and one experienced midwife, all of whom are employed at Oslo University Hospital but are unrelated to and independent of the study group members and principal investigator (PI). All separate primary outcome events will be presented to the DAG, who will discuss and evaluate whether the events are related to the study or not, and whether the events could have been prevented or not.

**Table 2:** Description of the clinical outcome variables of the HOME study.

Variable category	Variable	Definition/description	Evaluated by the Diagnostic Advisory Group
Primary composite outcome	Maternal death (direct)	Maternal death during pregnancy until 42 days postpartum due to pregnancy complications	Yes
	Severe maternal morbidity/organ damage	• Sepsis • Eclampsia • Cerebral hemorrhage • Acute respiratory distress syndrome • Liver rupture • Pulmonary embolism • Amniotic fluid embolism • HELLP (hemolysis, elevated liver enzymes, low platelets)/ELLP (HELLP without hemolysis) • DIC (disseminated intravascular coagulation)	
	Fetal/neonatal death	Intrauterine fetal death or death first 4 weeks after birth	
Secondary maternal outcomes Secondary offspring outcomes	Chorioamnionitis	Clinical diagnosis	No
	Malignant hypertension	Persistent systolic blood pressure ≥160 mmHg and/or diastolic blood pressure ≥100 mmHg	
	Adverse neonatal outcome	*Apgar* score <7 at 5 min of age *Umbilical cord acidemia* (arterial pH ≤7.10) *Metabolic acidosis* (umbilical artery pH <7.00 combined with base deficit in the extracellular fluid ≥12 mmol/L) (18)	
	Neonatal intensive care unit	Days	
	Respirator/ventilation support treatment	Days	

The secondary study outcomes (Table 2) include maternal morbidity [chorioamnionitis and malignant hypertension (persistent systolic blood pressure ≥160 mm Hg and/or diastolic blood pressure ≥100 mmHg)] and adverse neonatal outcomes (Apgar score <7 at 5 min, cord acidemia, metabolic acidosis (18), admission to neonatal intensive care unit, and/or respirator/ventilation support treatment). Umbilical cord acid-base data will be validated according to the method described by Kro et al. (19, 20).

In addition to the daily reports, the patient-reported outcome measures in the HOME study will be collected by digital questionnaires sent to the study participants' smartphones 2–7 days after hospital admission or start of home monitoring, and again at 10–12 weeks postpartum, for study group comparison. One digital reminder will automatically be sent to non-responders. The validated questionnaires include the Bergen Insomnia Scale (21), the General Perceived Self-Efficacy Scale (22), Sense of Coherence-13 (23), Edinburgh Postnatal Depression Scale (24–26), and the 25-item version of the Hopkins Symptom Checklist (27, 28). In addition, explanatory variables for multivariable analyses will be added, including a history of depression (based on DSM-IV criteria) (26, 29) and major life events during the previous 12 months, including serious events related to marriage, family, friends, work, or health (26), selected from established life event scales (26, 30, 31). In addition, a few HOME study-specific questions/items were constructed for the particular study that are not validated. After completing the questionnaires, the participants will be informed about available help resources if they are in need of further mental support during a potentially demanding period in their life (e.g., from their primary healthcare provider or a Norwegian mental health organization that is available 24/7).

The cost of home monitoring will be compared to hospital monitoring, including the maternal costs until delivery, but excluding maternal and newborn healthcare costs after delivery. The health economics assessment will evaluate hospital costs, including costs related to health professionals and administrative personnel time, for both outpatient and inpatient care. The mean cost per outpatient consultation and inpatient hospital cost per day will be calculated. The estimated mean cost is multiplied by the number of registered contacts at different departments during the study time (until delivery). Using the overhead indirect cost method, we will calculate the average estimated mean cost for different wards where the patient has a registered contact at the Oslo University Hospital. All costs will be evaluated as if operating under steady-state conditions based on the budget of 2023. Prices will be converted from Norwegian crowns (NOK) to U.S. dollars (US$) using the mean exchange rate for 2023 of 1 US$ = 9.86 NOK. All patient costs will be covered by the Norwegian public insurance system.

### Add-on placenta biomarker and CTG study

The development of improved surveillance tools to detect intrapartum or antepartum fetal stress is needed to prevent neonatal adverse outcomes, as CTG has limitations. We have previously found that predelivery circulating maternal antiangiogenic protein concentrations, produced by the placenta, may improve automated alerts produced by the Oxford System for computerized intrapartum monitoring (OxSys) 1.7 prototype (32). Depending on the technical feasibility, a predelivery placenta-associated biomarker and computerized antepartum and intrapartum fetal heart rate pattern study is planned in collaboration with UK researchers using data from the women in the HOME study.

### Sample size

The sample size calculation is based on the non-inferiority design and will be calculated for the primary clinical composite outcome (described above and in Table 2). The non-inferiority margin was set to 10% (i.e., a difference larger than 10% will be considered clinically significant), based on careful clinical considerations. The expected proportion of the outcome was set to 10%, based on the rate of the primary composite outcome observed in the first 80 women recruited to the hospital-monitored group in the HOME study. If there is no true difference between the home-monitored and the hospital-monitored groups' primary composite outcomes (10% in both groups), a total of 224 women with high-risk pregnancies (112 women in each group) is required. With this number of participants, we have a power of 0.80, ensuring that the upper limit of a one-sided 95% confidence interval will exclude a difference in favor of the hospital-monitored group of more than 10%. An additional six women will be invited to take potential dropouts into account. Further, due to the non-parallel group design (women were not randomized to either group, but are first mainly allocated to the hospital-monitored group and then to the home monitored group, pending on availability of home monitoring resources and equipment), another 20 women will be recruited to adjust for potential confounding factors such as parity and gestational age (totalling 250; 125 women in each group).

### Data analysis

After inclusion in the study, baseline data such as patient demographics, medical and obstetric history, and pregnancy data will be collected and registered in a case report form before entering and coding the data in a study-specific electronic record. After delivery, the remaining relevant data for the assessment of maternal mortality and morbidity and perinatal outcomes will be collected from the hospital's electronic patient journal and registered in the study-specific HOME records. All primary and secondary outcome data will be registered, in addition to gestational age at birth and birthweight (Table 2).

The composite primary outcome will be analyzed using logistic regression analyses. Parity and gestational age will be included as potential confounding variables. Further, each component of the composite primary outcome and all secondary outcomes will be analyzed to provide further insight into the research question. The results will be presented as odds ratios with 95% confidence intervals, in addition to crude proportions (not adjusting for parity and gestational age).

The obstetric experts in the DAG will independently evaluate all composite primary outcomes continuously throughout the project period and without interference from anyone in the project group. The DAG will conclude whether any primary (severe adverse) outcomes are related to the study or not, and the results will be presented in detail in the study report.

The study participants' experience, mental wellbeing (depression and anxiety), sense of coherence, and self-efficacy will be described at the group level (hospital monitoring vs. home monitoring) at the two investigated time points (2–7 days after hospital admission or start of home monitoring and again at 10–12 weeks postpartum).

The cost-effectiveness of home monitoring compared to the hospital-monitored group will be described at the group level based on the estimated cost of each admission to different wards at Oslo University Hospital and outpatient visits from inclusion in the HOME study until delivery.

The generalizability of the study sample will be evaluated by comparing the study group characteristics with the general pregnant population, the entirety of the latter being registered due to the compulsory notification of each birth to the Medical Birth Registry of Norway (33). Only women with Norwegian language skills can participate, which excludes some groups, particularly a subgroup of newly immigrated women, limiting the generalizability of the study. The present study can, however, be considered a first step in providing more personalized and flexible follow-up for women with high-risk pregnancies. If the study results are reassuring, future projects may include more heterogeneous language and ethnic groups, thus testing home monitoring opportunities in more diverse pregnant populations.

## Discussion

The HOME study will investigate whether home monitoring of high-risk pregnancies is non-inferior to inpatient hospital care regarding severe maternal and fetal adverse health outcomes, acceptability for users, and economic costs. Real-world prospective data will provide evidence of the feasibility, medical adverse outcomes (non-inferiority or not), patient-reported outcomes, and healthcare economics in a relatively homogeneous population in a high-income setting with a free-of-charge antenatal and obstetric care system for the patients.

For safety, a study-independent Diagnostic Advisory Group will regularly assess study adverse health outcomes and evaluate whether any primary severe adverse events are related to the study or not.

A limitation of the study is that recently immigrated women to Norway will likely be underrepresented in this study due to the need for adequate language skills. If this study finds that a telemonitored home healthcare service is non-inferior to in-hospital care, language support for recently immigrated women should be included with the aim of providing equal access to personalized care.

Recent technological development has generated new possibilities for healthcare at home for several patient groups, including pregnant women with high-risk pregnancies. We have created a new home follow-up program, with the goal of offering home-oriented and personalized care for women who need intensified surveillance in selected high-risk pregnancies. The aim of this study was to assess the effect of this program in a non-inferiority study design. The results may affect pregnancy monitoring practices and provide the knowledge needed to determine the safety, patient experience, and cost of implementing a home healthcare service in hospitals in Norway and other countries with similar healthcare systems.
