# Supplementary material for: The HOME (home monitoring of high-risk pregnancies) study: a study protocol for an observational study of a telemedicine-assisted follow-up at home vs. hospitalization
Source: Front Glob Womens Health. 2025 Jul 14;6:1599153. doi: 10.3389/fgwh.2025.1599153 (PMC12301385; doi:10.3389/fgwh.2025.1599153)
Supplement: Supplementary file 1 [file Table1.docx]

Questions Dignio (translation from the Norwegian version to English)

All patient registrations should be submitted through the MyDignio-app by 10:00 a.m

--------------------------------------------------------------------------------------------------

Questions for the pPROM (preterm premature rupture of membranes) group of home telemonitored women

- Does the baby in your womb move as usual?
  - Yes ______________Green response
  - No ______________ New question
- Didn't move for hours ______________Red response/call the hospital
- Less movement than before ______________Yellow response/call the hospital
- Do you feel sick/unwell and/or have a fever?
  - No ______________Green
  - Yes ______________ Red response/call the hospital
- Do you feel pain/discomfort/tenderness in your belly?
  - No ______________Green
  - Yes ______________Red response/call the hospital
- Have you noticed changes in the smell and/or color of the amniotic fluid?
  - No ______________Green
  - Yes ______________Red response/call the hospital
- Have you had fresh vaginal bleeding since the last report?
  - No ______________Green
  - Yes ______________ Red response/call the hospital

| Parameter | Values | Alarm colour on the hospital dashboard |  |
| --- | --- | --- | --- |
| Temperature (^o^C) | ≤37.5 | Green |  |
|  | 37.6-37.9 | Yellow |  |
|  | ≥38.0 | Red |  |
| Maternal pulse (per minute) | ≤50 | Yellow |  |
|  | 51- 99 | Green |  |
|  | ≥ 100 | Red |  |
| CRP in blood, measured and entered by the patient | ≥ 10 | Red |  |
| Questionnaire: subjective symptoms | ≥ 1 Red answer | Red |  |

Colour codes for the various response alternatives given by the home monitored pPROM patients, visible at the hospital for home monitored patients

Questions for the hypertensive disorders of pregnancy group of home telemonitored women

- Does the baby in your womb move as usual?
  - Yes ______________Green response
  - No ______________ New question
- Didn't move for hours ______________Red response/call the hospital
- Less movement than before ______________Yellow response/call the hospital
- Do you have a strong headache?
- No _______________ Green
- Yes ___________
  - (If yes:) Is the headache gone after taking pain relief (such as Paracetamol)?
- Yes _______________ Green
- No _______________ Red response/call the hospital
- Do you have visual disturbances and/or see flashes of light and/or flickering lights in the eyes?
- No _______________ Green
- Yes _______________ Red response/call the hospital
- Have you been short of breath?
- No _______________ Green
- Yes _______________ Red response/call the hospital
- Have you felt pain/pressure/a feeling of pressure in the upper part of the belly/under the rib cage?
  - No _______________ Green
  - Yes _______________ Red response/call the hospital
- Do you feel unwell or nauseous?
- No _______________ Green
- Yes _______________ Red response/call the hospital
- Do you have increasing and large swellings in your ankles, hands or face?
- No _______________ Green
- Yes _______________ Red response/call the hospital

Colour codes for the various response alternatives given by the home monitored patients with hypertensive diseases of pregnancy, visible at the hospital

| Parameter | Values | Alarm colour on the hospital dashbord |  |
| --- | --- | --- | --- |
| Blood pressure (mmHg) | Systolic <140 | Green |  |
|  | Systolic 140-149 | Yellow |  |
|  | Systolic ≥150 | Red |  |
|  | Diastolic <90 | Green |  |
|  | Diastolic 90-99 | Yellow |  |
|  | Diastolic ≥100 | Red |  |
| Urine dipstix | Protein 0 or traces of protein | Green |  |
|  | Protein ≥ +1 | Yellow |  |
| Questionnaire: subjective symptoms | ≥ 1 Red answer | Red |  |

Questions for the previous adverse obstetric outcome group of home telemonitored women

- Does the baby in your womb move as usual?
  - Yes ______________Green response
  - No ______________ New question
- Didn't move for hours ______________Red response/call the hospital
- Less movement than before ______________Yellow response/call the hospital
- Do you have a strong headache?
- No _______________ Green
- Yes ___________
  - (If yes:) Is the headache gone after taking pain relief (such as Paracetamol)?
- Yes _______________ Green
- No _______________ Red response/call the hospital
- Do you have visual disturbances and/or see flashes of light and/or flickering lights in the eyes?
- No _______________ Green
- Yes _______________ Red response/call the hospital
- Have you been short of breath?
- No _______________ Green
- Yes _______________ Red response/call the hospital
- Have you felt pain/pressure/a feeling of pressure in the upper part of the belly/under the rib cage?
  - No _______________ Green
  - Yes _______________ Red response/call the hospital
- Do you feel unwell or nauseous?
- No _______________ Green
- Yes _______________ Red response/call the hospital
- Do you have increasing and large swellings in your ankles, hands or face?
- No _______________ Green
- Yes _______________ Red response/call the hospital

Colour codes for the various response alternatives given by the home monitored patients with previous adverse obstetric outcomes, visible at the hospital

| Parameter | Values | Alarm colour on the hospital dashbord |  |
| --- | --- | --- | --- |
| Blood pressure (mmHg) | Systolic <140 | Green |  |
|  | Systolic 140-149 | Yellow |  |
|  | Systolic ≥150 | Red |  |
|  | Diastolic <90 | Green |  |
|  | Diastolic 90-99 | Yellow |  |
|  | Diastolic ≥100 | Red |  |
| Urine dipstix | Protein 0 or traces of protein | Green |  |
|  | Protein ≥ +1 | Yellow |  |
| Questionnaire: subjective symptoms | ≥ 1 Red answer | Red |  |
